# Severity of pulmonary emphysema and lung cancer: analysis using quantitative lobar emphysema scoring: Erratum

**DOI:** 10.1097/MD.0000000000006070

**Published:** 2017-01-27

In the article “Severity of pulmonary emphysema and lung cancer: analysis using quantitative lobar emphysema scoring”,^[1]^ which appeared in Volume 95, Issue 48 of *Medicine*, the first affiliation was originally given incorrectly. Affiliation “a” should read as follows:

Department of Radiology, Gyeongsang National University School of Medicine, Jinju, Korea.

## References

[1] Bae K Jeon KN Lee SJ Severity of pulmonary emphysema and lung cancer: analysis using quantitative lobar emphysema scoring. *Medicine*. 2016 95:e5494. 27902611 10.1097/MD.0000000000005494 PMC5134818

